# Expanding clinical variability in FBXW7-related neurodevelopmental disorder: a multicenter case series

**DOI:** 10.1186/s11689-026-09705-0

**Published:** 2026-07-10

**Authors:** Salvatore Savasta, Francesco Fabrizio Comisi, Giovanni Battista Dell’Isola, Gabriele Di Pasquale, Ivy Johnson, Isabella Herman, Andrea Maria Comisi, Francesca Felicia Operto, Giuditta Bargiacchi, Daniëla Q.C.M. Barge-Schaapveld, Giuseppe Donato Mangano, Marco Carotenuto, Vincenzo Salpietro, Alberto Verrotti

**Affiliations:** 1 Pediatric Clinic and Rare Diseases, Microcitemico Hospital “A. Cao”, University of Cagliari, Cagliari, Italy; 2 Department of Medical Sciences and Public Health, University of Cagliari, Cagliari, Italy; 3 Department of Medicine and Surgery, Saint Camillus International University of Health Sciences, Rome, Italy; 4 Department of Developmental Disabilities, IRCCS San Raffaele Roma, Rome, Italy; 5 Department of Biotechnological and Applied Clinical Sciences, Academic Unit of Pediatrics, University of L’Aquila, L’Aquila, Italy; 6 Munroe-Meyer Institute for Genetics and Rehabilitation, University of Nebraska Medical Center, Omaha, NE USA; 7 Boystown National Research Hospital, Boystown, NE USA; 8 Department of Clinical and Experimental Medicine, University of Catania, Catania, 95131 Italy; 9 Institute of Neurology, Department of Health Sciences Centre for Research in Unusual Infections, Epilepsy and Neuroscience (CRUISE), Magna Graecia University, Catanzaro, 88100 Italy; 10 Clinic of Child and Adolescent Neuropsychiatry, Department of Mental Health, Physical and Preventive Medicine, University of Campania “Luigi Vanvitelli”, Caserta, Italy; 11 Department of Clinical Genetics, Leiden University Medical Centre, Leiden, The Netherlands; 12 Department of Medicine and Surgery, University of Enna Kore, Enna, Italy; 13 European Brain Research Institute “Rita Levi-Montalcini” Viale Regina Elena, Rome, Italy; 14 Department of Pediatrics, University of Perugia, Perugia, Italy

**Keywords:** FBXW7, FBXW7-Related Disorder, Neurodevelopmental Disorders, Developmental Delay, Intellectual Disability, Variable expressivity

## Abstract

**Background:**

Heterozygous variants in *FBXW7* have recently been recognized as a cause of a rare neurodevelopmental disorder with variable developmental delay, neurological manifestations, and multisystem involvement. The breadth of clinical variability and penetrance remains incompletely defined.

**Cases presentation:**

We report a retrospective multicenter case series of seven previously unreported individuals (five males, two females) with heterozygous *FBXW7* variants identified through clinical genetic testing, aged 5–9 years at last evaluation (median 6 years). Six variants occurred de novo and one was inherited. Neurodevelopmental involvement was present in six individuals and was characterized by global developmental delay and language impairment; hypotonia was observed in all seven. Formal intellectual disability was documented in four cases, while one individual showed preserved cognitive functioning with predominant behavioral difficulties. Epileptic seizures occurred in four individuals, whereas three had no history of epilepsy. Brain MRI was available for six individuals and was normal in four, whereas two showed structural anomalies involving the corpus callosum. Extracerebral features were variably reported, most commonly constipation and recurrent respiratory/otolaryngological infections. Comparison with previously reported individuals confirmed the core neurodevelopmental phenotype and further refined the spectrum.

**Conclusions:**

This case series expands the phenotypic spectrum associated with *FBXW7*-related neurodevelopmental disorder and highlights variable expressivity and incomplete penetrance, including clinically relevant variants presenting with mild or atypical phenotypes. These findings support considering *FBXW7* across a broad range of neurodevelopmental presentations and inform genetic counseling.

**Supplementary Information:**

The online version contains supplementary material available at 10.1186/s11689-026-09705-0.

## Introduction

**Fig. 1:**
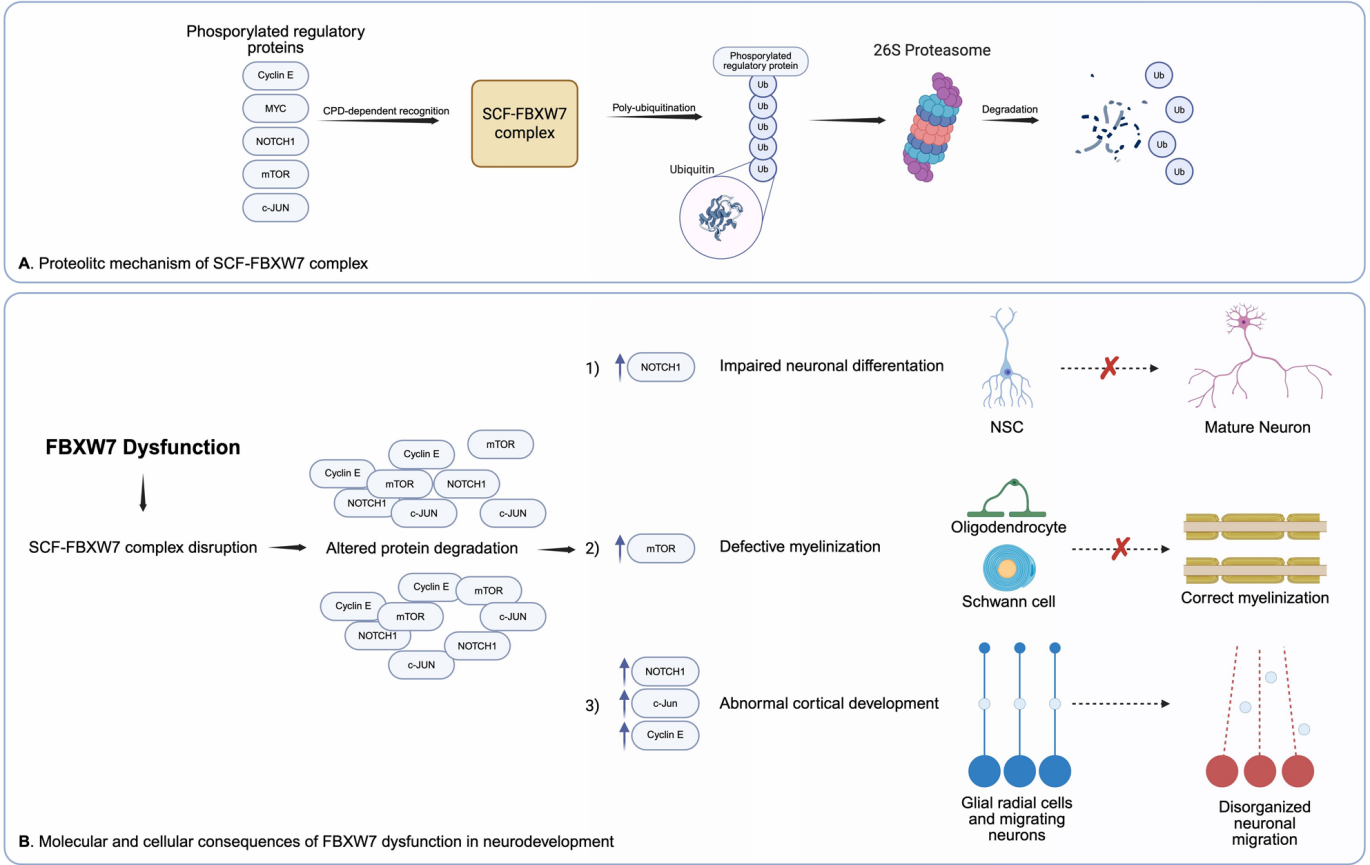
Molecular and Cellular Mechanisms of FBXW7-Related Neurodevelopmental Disorder. **A** Proteolytic mechanism of the SCF-FBXW7 E3 ubiquitin ligase complex. Phosphorylated regulatory proteins (Cyclin E, MYC, NOTCH, mTOR, c-JUN) are recognized by FBXW7 through CPD-dependent binding. The SCF-FBXW7 complex catalyzes poly-ubiquitination of target substrates, marking them for proteasomal degradation via the 26S proteasome. Ub, ubiquitin. **B** Molecular and cellular consequences of FBXW7 dysfunction in neurodevelopment. Loss of FBXW7 function disrupts the SCF-FBXW7 complex, leading to impaired ubiquitin-mediated protein degradation and accumulation of key regulatory substrates. This results in three major neurodevelopmental defects: (1) Impaired neuronal differentiation, elevated NOTCH signaling disrupts the transition from neural stem cells (NSC) to mature neurons; (2) Defective myelination, dysregulated mTOR signaling impairs oligodendrocyte and Schwann cell function, preventing proper axonal ensheathment; (3) Abnormal cortical development, dysregulation of NOTCH, c-Jun, and Cyclin E disrupts radial glial scaffold integrity and neuronal migration, resulting in cortical dyslamination. Red X symbols indicate blocked or impaired processes. Blue structures represent normal developmental scaffolds; red structures indicate disrupted pathways in FBXW7-related disease. Created in BioRender. Comisi, A. (2025) https://biorender.com/2yut15y

Variants affecting genes involved in ubiquitin-mediated protein degradation have increasingly been implicated in human neurodevelopmental disorders (NDDs). *FBXW7* encodes the F-box and WD-repeat-domain-containing 7, the substrate-recognition component of the SCF (SKP1-CUL1-F-box) E3 ubiquitin ligase complex, a central regulator of protein turnover through the ubiquitin-proteasome system, controlling the stability of multiple substrates involved in cell cycle regulation, differentiation, and developmental signaling pathways [1–5]. Although *FBXW7* has been extensively characterized as a tumor suppressor gene, with somatic alterations reported across a wide range of human malignancies [3, 4, 6–8], its role in brain development has only recently been recognized. Experimental studies have demonstrated that *FBXW7* is required for neuronal differentiation, cortical development, myelination, and cerebellar maturation, acting through key pathways including Notch, c-Jun, and mTOR signaling [2, 5, 9–17]. These observations provide a strong biological rationale for the involvement of *FBXW7* dysfunction in neurodevelopmental phenotypes. Experimental studies have demonstrated that FBXW7 regulates key substrates involved in neurogenesis, oligodendrocyte differentiation, and CNS myelination, including NOTCH, CYCLIN E, and Myelin Regulatory Factor [18, 19]. Disruption of these pathways provides a plausible molecular substrate for the neurodevelopmental phenotype and the white matter and corpus callosum abnormalities observed in affected individuals. The molecular mechanisms underlying these processes are illustrated in Fig. 1. The first systematic association between heterozygous *FBXW7* variants and a neurodevelopmental phenotype was established in 2022, when Stephenson et al. described a cohort of 35 individuals presenting with developmental delay (DD), hypotonia, language impairment, epilepsy, and variable neuroradiological abnormalities [20]. This study defined FBXW7-related NDD as an autosomal dominant condition, displaying a wide range of phenotypic features. Subsequent reports have expanded the clinical spectrum through additional single cases and small series, including individuals with copy number variants involving the 4q31.3 locus [21] and cases with milder or atypical presentations [22–25]. Despite these advances, the phenotypic landscape of FBXW7-related NDD is not yet fully established. Recent descriptions of Wilms tumor arising in individuals with heterozygous *FBXW7* variants, often in association with somatic second-hit events, have raised questions regarding cancer predisposition and clinical surveillance in this population [23, 25, 26]. In this context, the systematic reporting of additional well-characterized individuals is essential to refine the clinical spectrum, explore genotype-phenotype relationships, and improve diagnostic recognition. We herein present a multicenter case series of seven previously unreported individuals with heterozygous *FBXW7* variants identified through clinical genetic testing and discuss their clinical features in comparison with previously reported cases.

## Materials and methods

### Study design and participants

This study is a retrospective multicenter case series including children with heterozygous *FBXW7* variants identified through clinical genetic testing. Patients were referred for genetic evaluation because of neurodevelopmental concerns and were included following the detection of an *FBXW7* variant considered relevant in the clinical diagnostic context. No additional inclusion or exclusion criteria were applied.

### Clinical data collection

Clinical data were collected retrospectively from available medical records and included perinatal history, developmental milestones, neurological manifestations, seizure history, neurodevelopmental assessments, neuroimaging findings, extracerebral features, and available follow-up information. Neurodevelopmental evaluation was performed according to local clinical practice and encompassed assessment of cognitive, behavior, language and motor domains. Brain magnetic resonance imaging (MRI) and electroencephalography (EEG) investigations were reviewed when available across the cohort.

### Genetic analysis

Genetic testing was performed as part of routine diagnostic work-up through whole-exome sequencing (WES) at each referring center. Variants were annotated according to Human Genome Variation Society (HGVS) nomenclature at the coding DNA and protein levels. Variant interpretation and classification were performed in accordance with the American College of Medical Genetics and Genomics guidelines [27]. Parental testing was performed when available to assess inheritance.

### Literature review

A targeted review of the literature was conducted to identify previously reported individuals with *FBXW7* variants and neurodevelopmental phenotypes. Relevant studies were retrieved from peer-reviewed publications and used for qualitative comparison with the present cohort. PubMed, OMIM and ClinVar were searched from inception to January 2026 using the terms “FBXW7” combined with “neurodevelopmental disorder”, “developmental delay”, “intellectual disability”, “hypotonia”, “macrocephaly” and “Wilms tumor”; reference lists of retrieved articles were hand-screened for additional cases. Reports were included if they described individuals carrying heterozygous germline *FBXW7* variants with neurodevelopmental or multisystem phenotypes; somatic variants identified in tumors only were excluded. For each individual, clinical features reported as explicitly assessed and absent were coded as “absent”, whereas features not reported or not evaluated were coded as “not assessed” (NA); for this reason, denominators in Table 1 vary across features and reflect the number of individuals in whom each item was ascertainable from the source publications.

**Table 1:** Demographic and clinical features of individuals with pathogenic FBXW7 variants

Feature	Value	Feature	Value
**Demographic features**		**Ophthalmologic features**	
Sex	37 male / 12 female	Strabismus	7/44
Age range	2 months − 44 years, 6 months	Abnormality of refraction	6/44
**Medical history**		Ptosis	3/44
**Prenatal history**	Generally normal; isolated premature birth reported	Cerebral visual impairment	1/44
**Development, cognition, and psychiatric features**		Astigmatism	1/44
Neurodevelopmental abnormality	48/49	**Cardiac features**	
GDD or ID	40/49	Abnormal heart morphology	10/46
Delayed speech and/or language impairment	31/49	Septal defects (VSD; atrial defect)	4/46
Specific learning disability	2/49	Valve anomalies (bicuspid aortic valve; mildly dysplastic tricuspid aortic valve)	3/46
Autism spectrum disorder	3/49	Other vascular/patency anomalies (PDA surgery; persistent left superior vena cava to coronary sinus)	2/46
No neurodevelopmental abnormality	1/49	Interrupted aortic arch with subaortic stenosis	1/46
**Neurologic or CNS features**		**Respiratory features**	
Hypotonia	32/49	Recurrent respiratory infections or pneumonia	6/46
Abnormality of brain morphology	18/27	Congenital diaphragmatic hernia	1/49
Seizures	13/49	**Audiology and hearing**	
Macrocephaly	16/49	Mixed hearing impairment	2/42
Microcephaly	2/49	**Oral**,** dentition**,** and other ENT features**	
Hypertonia	3/49	Abnormal palate or uvula morphology	10/37
Ataxia	3/49	Laryngeal cleft	1/42
Developmental regression	1/49	Gastrointestinal and feeding features	
Craniofacial and dysmorphic features		Constipation	18/47
Any facial dysmorphism	45/48	Feeding difficulties	14/47
Deep-set eyes	11/48	Gastroesophageal reflux	8/47
High/arched/ogival palate	10/48	Intestinal malrotation	1/47
Broad/prominent forehead	8/48	Renal and genitourinary features	
Hypertelorism	7/48	Cryptorchidism	5/37 males
Epicanthal folds	7/48	Renal anomalies	3/45
Low/depressed nasal bridge	6/48	Hydrocele	2/37 males
Synophrys	4/48	Hematological and vascular features	
Small mouth	4/48	Neutropenia	2/42
Low-set ears	3/48	Anemia	1/42
Cleft palate	3/48	Venous thrombosis	1/42
Thin upper lip	2/48	Malignancy	
Micrognathia/chin hypoplasia	2/48	Wilms tumor	2/49
Thick eyebrows	2/48		
Long palpebral fissures	1/48		
Downslanting palpebral fissures	1/48		
Facial asymmetry	1/48		

Features are reported as n/N; denominators vary according to data availability

Data compiled from Stephenson et al., 2022 (*n* = 35); Meier-Abt et al., 2024 (*n* = 1); Pande et al., 2024 (*n* = 1); Wang et al., 2024 (*n* = 1); Zhou et al., 2024 (*n* = 2); Saito et al., 2025 (*n* = 1); Kim and Lim, 2025 (*n* = 1); and our cohort (*n* = 7)

*Abbreviations: DD* developmental delay, *GDD* global developmental delay, *ID* intellectual disability, *CNS* central nervous system, *VSD* ventricular septal defect, *PDA* patent ductus arteriosus

### Ethical considerations

All clinical and genetic investigations were performed within the framework of standard diagnostic care. This retrospective study was based exclusively on anonymized clinical data collected from existing medical records and did not involve additional procedures or interventions beyond routine care.

## Results

### Case series overview

**Fig. 2:**
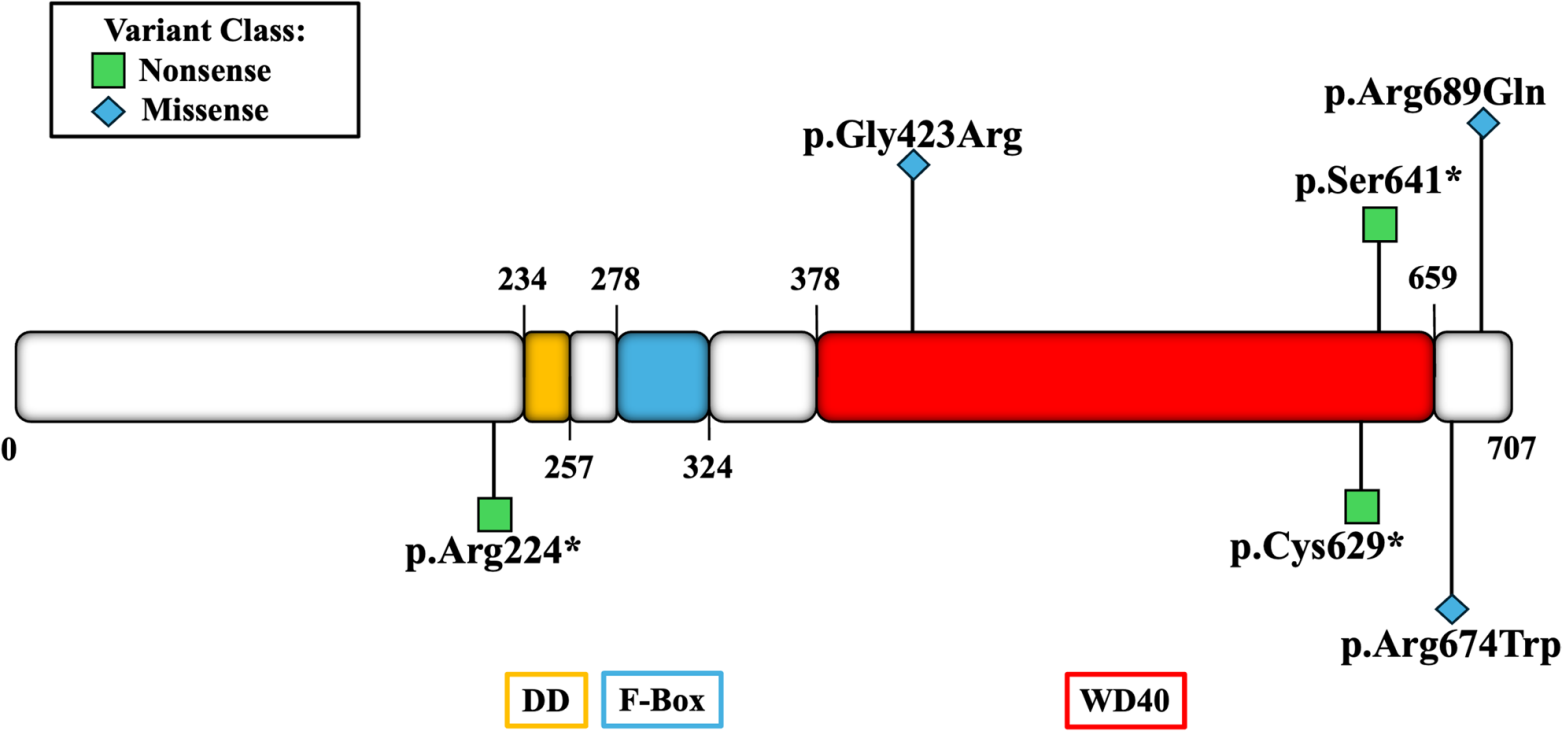
Distribution of FBXW7 variants identified in this study. Lollipop plot showing the position of variants along the FBXW7α protein (707 amino acids). Functional domains (DD, F-box-like, and WD40) are indicated. Variants cluster predominantly in the WD40 domain

**Fig. 3:**
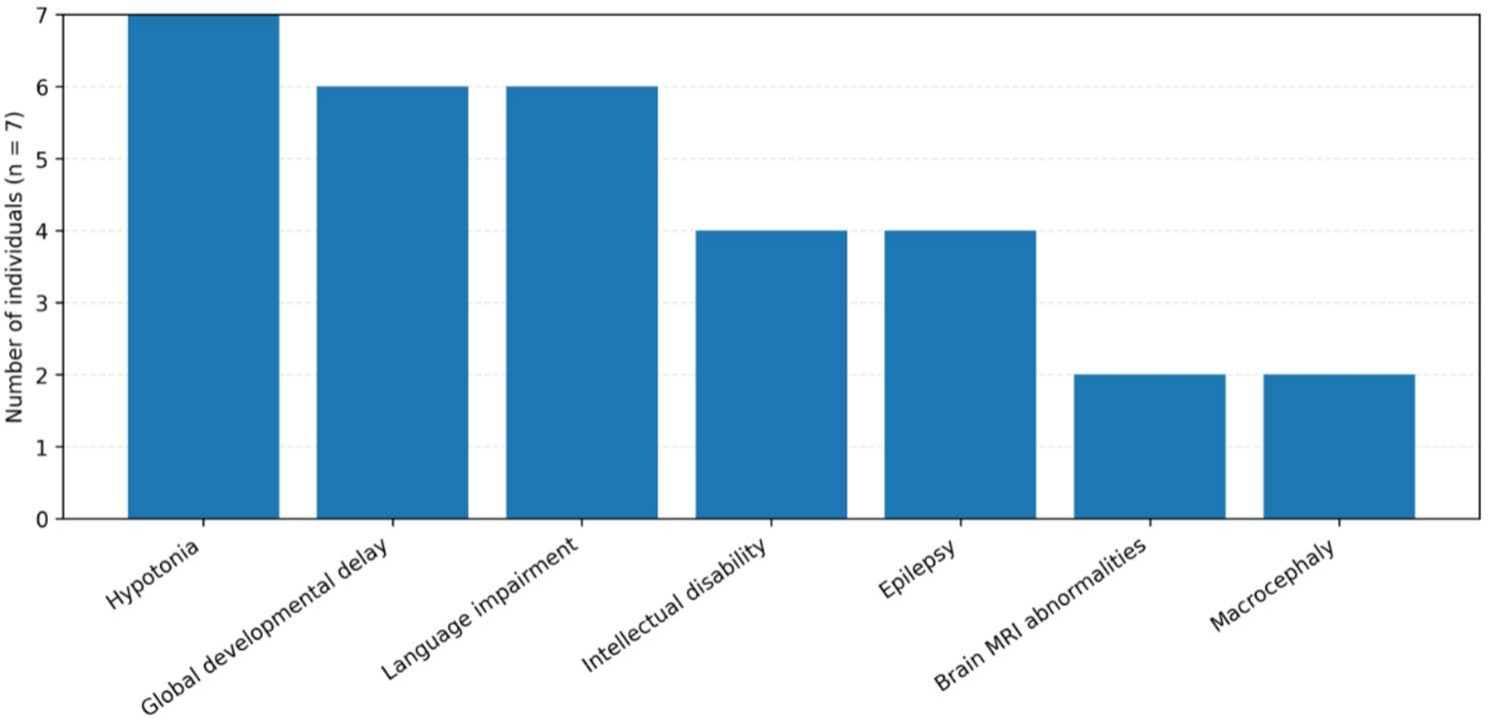
Distribution of main clinical features in the cohort. The bar chart summarizes the presence of key manifestations across the cohort, ordered from most to least frequent, based on available clinical data

The cohort comprised seven individuals (five males and two females), aged between 5 and 9 years at last clinical evaluation (median age: 6 years). Six individuals carried “*de novo” FBXW7* variants, whereas one harbored an inherited variant. The distribution of reported variants throughout *FBXW7* is shown in Fig. 2. Neurodevelopmental involvement was observed in six individuals and was characterized by global DD and language impairment. Hypotonia was present in all individuals. Formal intellectual disability (ID) was documented in four cases, while cognitive functioning was reported as preserved in one individual. Epileptic seizures were reported in four individuals, with heterogeneous seizure types and clinical courses, whereas three patients had no history of epilepsy. Brain MRI was available for six individuals and was normal in four; structural abnormalities were identified in two, both involving the corpus callosum. Head circumference was within the normal range in five individuals, consistent with macrocephaly in two. Extracerebral manifestations were variably reported, with constipation representing the most frequent associated feature, followed by recurrent respiratory or otolaryngological involvement. The distribution of the main clinical features observed in the cohort is summarized in Fig. 3, variant characterization and ACMG/AMP classification are presented in Table 2, while detailed case-level clinical and molecular data are provided in Additional File 1.

**Table 2:** Variant summary and ACMG/AMP classification of FBXW7 variants identified in this cohort

Case	Variant (NM_001349798.2)	Protein	Domain	Inheritance	gnomAD v4.1	ClinVar accession	ACMG/AMP criteria	Classification
1	c.1886_1887insATGCTGTGACCTG	p.(Cys629Ter)	WD40	De novo	Absent	Not submitted	PVS1, PS2, PM2	Pathogenic
2	c.670 C > T	p.(Arg224Ter)	-	De novo	Absent	VCV001722937 (Pathogenic)	PVS1, PS2, PM2	Pathogenic
3	c.2020 C > T	p.(Arg674Trp)	WD40	De novo	Absent	VCV001703001 (Pathogenic)	PS1, PS2, PM1, PM2, PP3	Pathogenic
4	c.2066G > A	p.(Arg689Gln)	WD40	De novo	6.8 × 10⁻⁷	VCV001702999 (Pathogenic)	PS1, PS2, PM1, PM2_Supporting, PP3	Pathogenic
5	c.2020 C > T	p.(Arg674Trp)	WD40	De novo	Absent	VCV001703001 (Pathogenic)	PS1, PS2, PM1, PM2, PP3	Pathogenic
6	c.1267G > A	p.(Gly423Arg)	WD40	De novo	Absent	VCV001702998 (Pathogenic)	PS1, PS2, PM1, PM2, PP3	Pathogenic
7	c.1922 C > G	p.(Ser641Ter)	WD40	Maternal	Absent	Not submitted	PVS1, PM2	Likely Pathogenic

Variants are annotated on transcript NM_001349798.2 (HGVS nomenclature). Classification follows ACMG/AMP guidelines [27]; criteria codes are defined therein. Allele frequencies are reported from gnomAD v4.1. '–' indicates variant not located within a defined functional domain. Abbreviations: DD, dimerization domain; WD40, WD40 repeat domain; HGVS, Human Genome Variation Society; ACMG/AMP, American College of Medical Genetics and Genomics / Association for Molecular Pathology

#### Case 1

A 5-year-old male was born at 39 weeks’ gestation via spontaneous vaginal delivery (birth weight 3,840 g; Apgar scores 6/7/9) to healthy, non-consanguineous parents. Generalized neonatal hypotonia with weak crying required Neonatal Intensive Care Unit admission, and feeding was complicated by oxygen desaturations. He showed global developmental delay (independent sitting at 15 months; walking at 18 months) with persistent language impairment, for which early neuropsychomotor rehabilitation and intensive speech therapy were initiated within the first year of life. Physical examination revealed macrocephaly (> 97th centile) and dysmorphic features (epicanthal folds, long eyelashes, saddle nose), two hypopigmented macules (left arm and left calf), umbilical hernia, bilateral hydrocele, second-third toe syndactyly, pes planus, and ligamentous laxity. From age 2 years, paroxysmal neurological events culminated in a brief generalized tonic-clonic seizure with urinary incontinence lasting < 3 min. Seizures were incompletely controlled on valproate, and levetiracetam was added. Serial EEGs showed diffuse slowing with paroxysmal abnormalities during wakefulness and sleep, with sporadic sleep-activated epileptiform discharges; brain MRI was normal. He experienced recurrent upper-airway infections, including otitis media and tonsillitis. Echocardiography demonstrated mild dysplasia of a tricuspid aortic valve. Laboratory testing showed elevated thyroid-stimulating hormone with normal free thyroxine. WES identified a heterozygous truncating *FBXW7* variant (NM_001349798.2: exon 14, c.1886_1887insATGCTGTGACCTG), predicted to introduce a premature termination codon (p.Cys629Ter)).

#### Case 2

A 6-year-old female presented with moderate global DD associated with hypotonia, in the absence of a contributory family history. Language acquisition was delayed, whereas neither epileptic seizures nor non-epileptic paroxysmal events were reported. Head circumference was consistent with macrocephaly. Dysmorphic features included myopathic facies and congenital unilateral ptosis. Cardiac assessment was unremarkable. Brain MRI showed no abnormalities. Cutaneous examination revealed a sacral dimple, with normal spinal MRI. Constipation represented the main gastrointestinal complaint. No recurrent respiratory infections, endocrine abnormalities, or tumor diagnoses were documented, and renal ultrasound performed after diagnosis was normal. Management consisted of speech and physical therapy. WES detected a “*de novo”* heterozygous *FBXW7* nonsense variant, NM_001349798.2:c.670 C > T, p.(Arg224Ter).

#### Case 3

An 8-year-old male, diagnosed at the age of 2 years, with no family members affected by the same condition, presented with moderate global DD and moderate ID. Language development was impaired. Epilepsy was reported, characterized by tonic-clonic seizures. Neurological examination revealed hypotonia. Head circumference was within the normal range. Physical examination showed facial dysmorphisms, including a small mouth, ogival palate, and deeply set eyes. No cardiac anomalies were identified. No limb, hand, or foot anomalies were reported. Brain MRI was normal. No skin abnormalities were observed. The medical history was notable for gastrointestinal involvement, specifically constipation, while no recurrent respiratory infections, endocrine abnormalities, or tumor diagnoses were reported. Treatment history included valproic acid and lamotrigine. Molecular genetic testing performed by WES identified a heterozygous “*de novo” FBXW7* variant, NM_001349798.2:c.2020 C > T, p.(Arg674Trp), the same recurrent variant identified in Case 5 and previously reported by Stephenson et al. (2022) in two unrelated individuals.

#### Case 4

A 7-year-old male was diagnosed at 3 years of age and had no family members affected by the same condition. His clinical phenotype was characterized by moderate global DD and moderate ID, with a documented Intelligence Quotient (IQ) of 68, together with delayed language development. Tonic-clonic seizures were present but were well controlled with valproic acid. Hypotonia was evident on neurological examination, and head circumference was within the normal range. Dysmorphic features included prominent forehead and hypertelorism. Cardiac evaluation and examination of the limbs and skin were unremarkable. Brain MRI demonstrated corpus callosum hypoplasia and delayed myelination. Feeding and gastrointestinal problems were not reported. The clinical history included recurrent respiratory infections, whereas endocrine abnormalities and tumor diagnoses were absent. Treatment consisted of valproic acid and neuropsychomotor rehabilitation. WES detected a “*de novo”* heterozygous *FBXW7* variant, NM_001349798.2:c.2066G > A, p.(Arg689Gln).

#### Case 5

A 5-year-old male, diagnosed at 3 years of age, presented with moderate global DD and moderate ID (IQ 65), associated with delayed language development. Epilepsy manifested as focal seizures with secondary generalization beginning at 4 years of age and was effectively controlled with levetiracetam. In addition, non-epileptic paroxysmal events were reported, consisting of NREM parasomnias (night terrors). Neurological examination showed hypotonia, while head circumference was within normal limits. Mild, non-specific facial dysmorphisms were observed, including a broad and high forehead and a low nasal root. No cardiac, limb, or cutaneous anomalies were identified. Brain MRI revealed mild corpus callosum dysgenesis and mild vermian hypoplasia. Gastrointestinal involvement included chronic constipation requiring osmotic laxatives and food selectivity, and recurrent respiratory infections were also reported. Endocrine abnormalities and tumor diagnoses were not observed. Management included levetiracetam, neuropsychomotor therapy, and speech therapy. WES identified a “*de novo”* heterozygous *FBXW7* variant, NM_001349798.2:c.2020 C > T, p.(Arg674Trp).

#### Case 6

A 6-year-old female was diagnosed at 3 years of age and had no affected relatives. She presented with moderate global DD and ID, although formal IQ assessment could not be completed because of limited cooperation. Language development was delayed. Epileptic seizures were not reported; however, brief non-epileptic paroxysmal arousals from deep sleep associated with transient stiffening were described. Neurological examination revealed hypotonia, and head circumference was within normal limits. Mild, non-specific facial dysmorphisms were present, including slightly ptotic eyelids. No cardiac or limb anomalies were identified. An involuting labial hemangioma was reported by the parents. Brain MRI was normal. Feeding and gastrointestinal issues included intermittent constipation and feeding selectivity. Recurrent respiratory infections, endocrine abnormalities, and tumor diagnoses were not reported. Management comprised neuropsychomotor therapy, intensive speech therapy, augmentative and alternative communication strategies, parent training, and laxatives as needed. WES revealed a “*de novo”* heterozygous *FBXW7* variant, NM_001349798.2:c.1267G > A, p.(Gly423Arg).

#### Case 7

A 9-year-old male was evaluated for behavioral difficulties, associated with mild motor delay in the context of mild hypotonia. Drooling was reported when he was tired. Cognitive functioning was described as normal, and he attended a regular school. Speech was characterized by a nasal quality. Medical history was notable for constipation. On physical examination, head circumference was 54.9 cm (1.2 SDS). Brachycephaly without evidence of craniosynostosis was observed, a broad forehead along with hypertelorism, downslanting palpebral fissures, a thin upper lip, and pes planus. No epilepsy or non-epileptic paroxysmal events were reported. Brain magnetic resonance imaging was not done. Cardiac screening (ECG, echocardiogram) was normal. He had recurrent otitis media that was managed with tympanostomy tube placement. Information regarding limb anomalies, skin findings, recurrent respiratory infections, endocrine abnormalities, tumor diagnoses and treatments were not reported. WES identified a heterozygous truncating variant in the *FBXW7* gene, NM_001349798.2:c.1922 C > G, p.(Ser641Ter), located within a WD40 domain. The variant was inherited from the patient’s mother, who was reportedly unaffected; however, formal neurodevelopmental assessment was not performed.

## Discussion

### Principal findings

In this study, we describe seven new individuals carrying heterozygous *FBXW7* variants, thereby expanding the currently available clinical dataset for FBXW7-related NDD. Overall, our findings corroborate the core phenotype previously reported, with global DD, language impairment, and hypotonia representing the most consistently observed clinical features, while epilepsy was identified in a subset of individuals, displaying heterogeneous seizure phenotypes and variable response to antiseizure therapy [20]. Neuroimaging findings span from structurally normal brain MRI to abnormalities involving the corpus callosum and posterior fossa, further emphasizing the neuroradiological heterogeneity associated with this disorder. Beyond the neurodevelopmental profile, several individuals exhibited recurrent extracerebral manifestations, most frequently gastrointestinal involvement, accompanied by variable impairment of additional organ systems. Notably, one individual presented with a markedly attenuated phenotype characterized by preserved cognitive functioning and predominant behavioral features in the context of an inherited variant, providing further support for variable expressivity and incomplete penetrance, in line with previous reports [20, 22–25, 28].

### Neurodevelopmental manifestations associated with FBXW7 variants

Although global DD and language impairment represent the most consistently reported manifestations of FBXW7-related NDD, increasing evidence supports a broad spectrum of neurodevelopmental severity. In the largest published cohort to date, most individuals exhibited mild-to-moderate DD or ID, while a minority showed either severe impairment or preserved cognitive functioning [20]. This variability extended beyond cognition to include motor development, seizure occurrence, and neuroradiological findings. The present case series closely mirrors this heterogeneity. Hypotonia emerged as a frequent feature, variably associated with delayed motor milestones and differing degrees of functional impact. Epilepsy, when present, was clinically heterogeneous, encompassing generalized tonic-clonic seizures, focal seizures with secondary generalization, and absence-like episodes, with variable response to antiseizure medications. This phenotypic diversity suggests that seizure susceptibility in FBXW7-related NDD is not uniform and may evolve over time. Similarly, brain MRI findings ranged from normal studies to structural abnormalities, most commonly involving the corpus callosum and posterior fossa structures, underscoring neuroradiological variability even among individuals with comparable clinical severity. The coexistence within the same cohort of individuals with epilepsy, hypotonia, and structural brain abnormalities alongside patients with minimal neurodevelopmental involvement highlights the limited prognostic value of single clinical features when considered alone. Notably, one individual in our cohort showed preserved cognitive functioning, attended mainstream schooling, and presented predominantly with behavioral difficulties and mild motor involvement. This presentation aligns with the mildest end of the phenotypic spectrum previously described and provides further support for variable expressivity and incomplete penetrance in FBXW7-related NDD [20]. Collectively, these observations reinforce the concept of a phenotypic continuum, in which milder or atypical presentations may be underrecognized, with important implications for diagnostic evaluation and genetic counseling.

### Variable expressivity and incomplete penetrance

The clinical heterogeneity observed in FBXW7-related NDD is best interpreted within the framework of variable expressivity and incomplete penetrance, rather than as discrete phenotypic subtypes. The coexistence of markedly different neurodevelopmental outcomes among individuals carrying heterozygous *FBXW7* variants, including the presence of minimally affected individuals, indicates that pathogenic variants may manifest across a wide range of clinical severity, from overt neurodevelopmental impairment to subtle or subclinical presentations. In this context, the identification of an individual with no overt cognitive impairment in the present series is not an isolated finding but rather reinforces observations previously reported in larger cohorts [20]. Together with the absence of a clear genotype-phenotype correlation, these data suggest that additional modifiers, genetic or environmental, likely contribute to phenotypic expression, although their nature remains undefined. From a clinical perspective, recognition of incomplete penetrance is particularly relevant, as it implies that *FBXW7* variants may be identified in individuals with atypical or attenuated phenotypes, expanding the diagnostic spectrum beyond classical presentations. This conceptual framework has important implications for variant interpretation, counseling, and expectations regarding disease course.

### Comparison with previously reported cases

Comparison of the present cohort with previously reported individuals confirms substantial overlap with the clinical spectrum already delineated for FBXW7-related NDD and further refines the boundaries of its multisystem involvement. Based on currently available literature, at least 42 individuals with FBXW7-related NDD have been reported to date [20, 22–25, 28, 29]; with the addition of seven new patients, the cumulative dataset increases to 49 individuals (Table 1), and the present series contributes 14.3% of all published cases.

#### Neurodevelopmental features

Neurodevelopmental involvement is nearly universal. Across the 42 previously reported individuals, neurodevelopmental abnormalities were documented in 41, with only one individual described as having no neurodevelopmental impairment [20]. Language impairment, ranging from speech delay to absent verbal communication, was explicitly reported in 25 individuals, while autism spectrum disorder (ASD) was noted in three [20]. Rett-like manifestations with stereotyped behaviors, bruxism, and irregular breathing were described in a single recent case, suggesting these features may occur at the extreme of the phenotypic spectrum [29]. In our cohort, global DD was observed in six of seven individuals, and ID was confirmed in four; language impairment was present in six. One patient (Case 7) showed preserved cognition with predominant behavioral difficulties and only mild motor delay, supporting variable expressivity within the disorder.

#### Neurological manifestations

Hypotonia is the most consistently reported neurological feature, documented in 25/42 previously reported individuals, whereas hypertonia was rare (2/42) [20, 22, 24]. Epilepsy/seizures were reported in nine patients, encompassing heterogeneous seizure phenotypes including generalized and focal seizures and febrile events [20, 29, 30]. Ataxia or ataxic gait was documented in three cases, and progressive spasticity in one [20]. In our series, hypotonia was present in all seven individuals, and epilepsy affected four. Seizures were controlled in three affected individuals, and non-epileptic paroxysmal events (including NREM parasomnias) were reported in two patients.

#### Neuroradiological findings

Brain MRI data were available for 21 of 42 previously reported individuals. Structural abnormalities were present in 16/21 (76.2%) and normal imaging in 5/21 [20, 22, 24, 29]. Corpus callosum anomalies represent the most frequently described midline finding and were documented in seven individuals in the largest cohort, with additional reports of cerebellar anomalies, delayed myelination, brainstem changes, and less common findings such as polymicrogyria, calcifications, or sequelae of intracranial hemorrhage [20, 22, 24]. In the present cohort, MRI was available for six individuals and was normal in four; abnormalities were identified in two, both involving the corpus callosum (with delayed myelination in Case 4 and mild vermian hypoplasia in Case 5) [19]. The lower frequency of neuroradiological abnormalities in our series likely reflects the combined effects of small sample size, ascertainment differences, and/or age-dependent detectability.

#### Electroencephalographic findings

Systematic EEG data remain limited in literature. EEG abnormalities were explicitly described in two previously reported individuals, including delayed maturation of electrical activity in a neonate and intermittent slowing with focal spikes in another case [24, 29]. In our cohort, EEG was available for one individual (Case 1) and showed diffuse slowing and paroxysmal abnormalities with sleep-activated epileptiform discharges. Although sparse, these observations suggest that EEG abnormalities may accompany the phenotype in a subset of patients and merit systematic assessment in future studies.

#### Craniofacial features

No pathognomonic facial gestalt has emerged. Craniofacial involvement appears frequent and heterogeneous. Across previously reported individuals, head-size anomalies are recurrent, with macrocephaly documented in 14/42 and microcephaly in 2/42. Periorbital/orbital findings are also common, particularly deep-set eyes (10/42), while cleft palate and/or high/arched/ogival palate morphology was described in 11/42, sometimes in association with dental malocclusion. Additional variably reported features include a broad/prominent forehead, epicanthal folds, hypertelorism, thick eyebrows/synophrys, depressed nasal bridge, small mouth, and micrognathia, and a minority of individuals were reported as having no dysmorphic features, underscoring variable expressivity [20, 22–25, 28, 29]. In our cohort, dysmorphic features were noted in all seven individuals. Head circumference was normal in five individuals and consistent with macrocephaly in two, while recurrent hypertelorism was observed in two (Cases 4 and 7). Other findings included epicanthal folds and saddle nose (Case 1), congenital ptosis and myopathic facies (Case 2), deep-set eyes with small mouth and ogival palate (Case 3), and mild non-specific dysmorphism with ptosis (Case 6). Overall, these data support a broad and non-specific craniofacial spectrum, in which combinations of head-size anomalies, orbital configuration, and palate morphology may facilitate clinical recognition but are not individually diagnostic.

#### Ophthalmological features

Ocular abnormalities were common among previously reported individuals, with strabismus in 6 and refractive errors in 6, while cerebral visual impairment and other isolated findings were rarely described [20, 25]. In our cohort, ophthalmological involvement was documented in two individuals, both with ptosis, and no strabismus or refractive errors were reported.

#### Cardiovascular involvement

Cardiac anomalies were documented in 9 previously reported individuals, including septal defects, patent ductus arteriosus (occasionally requiring intervention), valvular anomalies such as bicuspid aortic valve, and rare complex malformations [20]. In our cohort, cardiac evaluation was unremarkable in six individuals, while one patient (Case 1) showed a mildly dysplastic aortic valve on echocardiography, supporting variable but potentially clinically relevant cardiovascular involvement.

#### Respiratory and otolaryngological involvement

Recurrent pneumonia was reported in three previously described individuals, and neonatal respiratory distress requiring intubation was described in one individual with congenital diaphragmatic hernia [20, 23]. Mixed hearing impairment was reported in two individuals and laryngeal cleft in one [20]. In the present cohort, recurrent respiratory and/or otolaryngological infections were documented in three individuals, including recurrent upper-airway infections, otitis media, and tonsillitis (Case 1) and recurrent respiratory infections (Cases 4–5), infection susceptibility may be under recognized or variable across cohorts.

#### Gastrointestinal manifestations

Gastrointestinal features are frequent but heterogeneous. Across previously reported individuals, constipation was documented in 13/40 and feeding-related difficulties (including neonatal sucking/swallowing problems and broader feeding difficulties) in 12/40; gastroesophageal reflux was reported in 8/40, with gastrostomy placement in four individuals and Nissen fundoplication in one [20, 23]. In the present cohort, gastrointestinal involvement was documented in 5/7 individuals, driven mainly by constipation (5/7) and feeding selectivity (2/7); none required enteral feeding support or surgery.

#### Genitourinary features

Genitourinary anomalies were variably reported. Cryptorchidism was documented in 5/32 previously reported males, and renal anomalies were reported in three individuals, including multicystic or dysplastic kidney and nephromegaly [20, 23]. Hydrocele was reported in one individual [22]. In our cohort, genitourinary involvement was limited to bilateral hydrocele in one patient, and no renal anomalies or cryptorchidism were observed.

#### Other manifestations and tumor predisposition

Hematologic abnormalities included neutropenia in two individuals and mild anemia in one, while venous thrombosis was reported in a single individual with multiple congenital anomalies [18–20]. Wilms tumor was documented in two previously reported patients, with biallelic FBXW7 inactivation demonstrated in tumor tissue, supporting a biologically plausible but apparently low-penetrance cancer predisposition signal [23, 25, 26, 31]. No malignancies were observed in the present cohort up to date.

### Study limitations

Some considerations should be noted when interpreting the findings of this study. As expected for a rare condition, the number of individuals included is limited, which constrains the exploration of genotype-phenotype relationships. Clinical data were collected retrospectively, and the duration and depth of follow-up varied across individuals, reflecting real-world clinical practice in a multisite context. In addition, neurodevelopmental assessments and neuroimaging protocols were not fully uniform across all patients, which may have introduced some variability in phenotypic characterization. Functional studies were not performed for the identified variants; however, the genetic findings were interpreted within the framework of established molecular mechanisms and previously published data. Systematic EEG evaluation was not available for all individuals with epilepsy at the time of data collection, and prospective EEG assessment in future follow-up may further characterize the electroclinical profile of this condition. Regarding Case 7, the variant was inherited from the mother, who was reportedly unaffected; however, she did not undergo formal neurodevelopmental assessment, which tempers the strength of inferences about incomplete penetrance drawn from this single family. Systematic re-analysis of non-coding regions was not undertaken. Despite these considerations, the present series adds a meaningful number of new individuals to the existing literature and provides further insight into the phenotypic spectrum of FBXW7-related NDD. Larger, prospectively collected cohorts will be key to further delineate the clinical spectrum and to clarify factors contributing to phenotypic variability.

## Conclusions

This case series adds further evidence that FBXW7-related NDD represents a clinically heterogeneous condition whose presentation cannot be reliably inferred from variant type or position alone. The broad range of phenotypes observed, including attenuated presentations, supports a model of variable expressivity and incomplete penetrance and highlights the limitations of narrow, phenotype-driven diagnostic frameworks. From a clinical standpoint, these findings emphasize the importance of considering *FBXW7* variants across a wide spectrum of neurodevelopmental presentations and reinforce the value of genome-wide sequencing approaches in identifying affected individuals. Continued aggregation of well-characterized cases will be essential to refine disease boundaries and inform clinical interpretation.

## Supplementary Information

Additional file 1: Supplementary Table 1. Clinical, neurodevelopmental, and molecular features of individuals with FBXW7-related neurodevelopmental disorder. Detailed patient-level data for the study cohort, including sex, age at last evaluation, FBXW7 variant and variant origin, presence of global developmental delay, intellectual disability, language impairment, epilepsy, hypotonia, head circumference, brain MRI findings, and additional clinical features

## Data Availability

The datasets used and/or analysed during the current study are available from the corresponding author on reasonable request.

## Abbreviations

ASD: Autism spectrum disorder
CNS: Central nervous system
CPD: Cdc4 phosphodegron
DD: Developmental delay
EEG: Electroencephalography
ENT: Ear, nose and throat
FBXW7: F-box and WD-repeat-domain-containing 7
GDD: Global developmental delay
HGVS: Human Genome Variation Society
ID: Intellectual disability
IQ: Intelligence quotient
MRI: Magnetic resonance imaging
mTOR: Mechanistic target of rapamycin
NDD: Neurodevelopmental disorder
NREM: Non–rapid eye movement
NSC: Neural stem cell(s)
PDA: Patent ductus arteriosus
SCF: SKP1–CUL1–F-box (E3 ubiquitin ligase complex)
SDS: Standard deviation score
Ub: Ubiquitin
VSD: Ventricular septal defect
WES: Whole-exome sequencing
